# ORAL CONCURRENT SESSION 6: Prematurity and Newborn

**DOI:** 10.1002/pmf2.12015

**Published:** 2025-01-05

Abstracts 67 – 76

FRIDAY

January 31, 2025

1:30 PM – 4:00 PM

Aurora Ballroom CD

MODERATORS

Vincenzo Berghella, MD

Michelle Y. Owens, MD

